# Bacterial contamination on used face masks among nursing home healthcare personnel

**DOI:** 10.1017/ash.2023.130

**Published:** 2023-03-15

**Authors:** Madison Nightingale, Manali Mody, Alexander H. Rickard, Marco Cassone

**Affiliations:** 1 Division of Geriatric & Palliative Medicine, Michigan Medicine, Ann Arbor, Michigan; 2 Department of Epidemiology, University of Michigan School of Public Health, Ann Arbor, Michigan

## Abstract

**Objectives:**

Since the beginning of the COVID-19 pandemic, face masks have been worn by many in public areas and for prolonged periods by healthcare workers (HCWs). This may facilitate bacterial contamination and transmission to and from patients in nursing homes where clinical care areas with strict precautions and residential and activity areas are interconnected. We assessed and compared bacterial mask colonization in HCWs belonging to different demographic categories and professions (clinical and nonclinical) and among HCWs who had worn the mask for different periods of time.

**Design, setting and participants:**

We conducted a point-prevalence study of 69 HCW masks at the end of a typical work shift in a 105-bed nursing home serving postacute care and rehabilitation patients. Information collected about the mask user included profession, age, sex, length of time the mask was worn, and known exposure to patients with colonization.

**Results:**

In total, 123 distinct bacterial isolates were recovered (1–5 isolates per mask), including *Staphylococcus aureus* from 11 masks (15.9%) and gram-negative bacteria of clinical importance from 22 masks (31.9%). Antibiotic resistance rates were low. There were no significant differences in the number of clinically important bacteria among masks worn more or less than 6 hours, and there were no significant differences among HCWs with different job functions or exposure to colonized patients.

**Conclusions:**

Bacterial mask contamination was not associated with HCW profession or exposure and did not increase after 6 hours of mask wearing in our nursing home setting. Bacteria contaminating HCW masks may differ from those colonizing patients.

Face masks have been regarded as a mainstay item for protection against COVID-19 and other communicable diseases.^1^ They are frequently touched or adjusted with the hands^2^ and may come in contact with various surfaces and high-touch sites when taken off and on even briefly. This presents an opportunity for face masks to become contaminated with microorganisms and be a vehicle of contamination.^3,4^ Unlike before the pandemic, face masks are now worn universally and for long periods of time by healthcare workers (HCWs),^5^ which may contribute to contamination. Nursing homes have high rates of multidrug-resistant organisms (MDROs)^6^ and low PPE compliance.^7^ However, little attention has been given to face masks as a vehicle of contamination. Therefore, evaluating contamination of face masks may be of great interest, especially in this setting. We characterized bacterial colonization on used face masks in HCWs, and we assessed the presence of clinically important bacteria such as methicillin-susceptible and resistant *S. aureus* (MSSA and MRSA), vancomycin-susceptible and resistant enterococci (VSE and VRE respectively), *Klebsiella pneumoniae, Enterobacter* and other gram-negative bacteria (GNB) resistant to 1 or more antibiotics belonging to several major antibiotic classes. We also compared the species that colonized face masks (all bacteria as well as recognized potential pathogens) among HCWs belonging to different demographic categories and professions (including clinical and nonclinical), and among HCWs who had worn the mask for different periods of time.

## Methods

### Study design and data collection

A point-prevalence study was conducted in August 2021 on personnel at a 105-bed nursing home serving mostly postacute care and rehabilitation patients. All HCWs exiting the facility at the end of their work shift were eligible to participate, regardless of job or function, and they were met just outside the lobby of the building at the end of the HCW’s work shift utilizing a stand that we set up for this purpose. The stand was set up 3 times on 3 separate days. At the end of the third session, we determined that that most personnel willing to donate a mask had already done so; thus, adding additional collection windows would not have meaningfully increased our sample size. Information about the mask and the user was collected via a self-reported survey: profession, age, sex, length of time the mask was worn, whether the user had provided care to one or more patients with known MDRO colonization. To maximize the number of participants involved, we did not attempt to limit entries by category. All participants were wearing surgical face masks and were asked to put their face masks inside a sterile collection bag, and a new face mask was offered. The study was approved by the University of Michigan Institutional Review Board (no. HUM00200673).

### Sample processing and identification

Within 2 hours of collection, face masks were incubated at 36ºC with 50 mL brain-heart infusion broth in a sterile collection cup for 18–24 hours. Then 10 μL broth was plated on selective and differential plates. Morphotypes suggestive of *S. aureus* on mannitol salt agar were identified using catalase and coagulase tests (Staphaurex, Remel, Lenexa, KS). Methicillin resistance was established using cefoxitin disc diffusion on Mueller-Hinton agar.^8^ Morphotypes suggestive of VSE on bile-esculin agar, and VRE on bile-esculin agar with 6 μL/mL vancomycin, were confirmed with pyrrolidonyl arylamidase test (PYR; Becton-Dickinson, Franklin Lakes, NJ). GNB growing on MacConkey agar were identified using API 20E strips (Biomerieux, Marcy-L’Etoile, France). Resistance to ciprofloxacin, meropenem, tetracycline, erythromycin, gentamicin, trimethoprim/sulfamethoxazole and ceftazidime with and without clavulanic acid was established using disc diffusion.^8^

### Data analysis

We calculated the overall prevalence of colonization with MSSA, MRSA, VSE, VRE, and all GNB species among all face masks, as well as colonization with at least 1 organism. The percentage of antimicrobial resistant strains was calculated for each antibiotic, as well as the % of strains resistant to at least 1 antibiotic. Next, the prevalence of all organisms and of selected, clinically important pathogenic organisms (MSSA, MRSA, VRE, VSE, *K. pneumoniae*, *Enterobacter*, *E. coli*) was compared between HCWs wearing the mask for <6 hours versus >6 hours, as well as among different profession groups (clinical vs nonclinical), different age groups (18–29, 30–44, 45–54, ≥55 years), and finally among HCWs who reported interacting with known MDRO-colonized patients during the shift, versus those who did not. An unpaired *t* test was used to test statistical significance between groups.

## Results

### Study population

In total, 66 mask donations were obtained from HCWs in the span of 6 days. In 3 cases, 2 masks were donated by HCWs who had been wearing both during their shift, yielding a total of 69 masks. Moreover, 6.0% of the survey responses identified as male, and 27.3% were aged 30–44 years, whereas 24.2% were aged 45–54 years. Responses identified the donating HCW as belonging to the following categories: CENA/NA (22.7%); nurse (12.1%); environmental services or housekeeping (7.6%); physician, nurse practitioner, or physician assistant (1.5%); and other or administrative (37.9%). In 8 (12.1%) of 66 cases, the HCW reported treating a patient with a known MDRO while wearing their mask.

### Prevalence of bacterial contamination and antimicrobial resistance rates

The number of total isolates recovered from each mask ranged from 1 to 5 (including gram-positive organisms, and gram-negative organisms). Our data indicated high bacterial burden on masks, including substantial rates of contamination with clinically important pathogens. *Staphylococcus aureus* was identified on 11 masks (15.9%), and VSE was identified on 30 masks (43.5%) (Table 1). High rates of mask contamination were found for GNBs, with 81 total isolates (1.17 per mask on average). Notably, 22 masks (31.9%) were contaminated with clinically important pathogenic gram-negative bacteria, including 14.5% with *Klebsiella pneumoniae*, 13.0% with *Enterobacter*, and 4.3% with *Escherichia coli* (Table 1).

**Table 1.** Prevalence of Gram-Positive and Gram-Negative Bacteria on All Face Masks

Species	Total (N=69) No. (%)
**Enterococcaceae**	
*Enterococcus spp.*	31 (44.9)
Vancomycin susceptible	30 (43.5)
Vancomycin resistant	1 (1.5)
**Staphylococcaceae**	
*Stapylococus aureus*	11 (15.9)
Methicillin susceptible	11 (15.9)
Methicillin resistant	0 (0.0)
**Enterobacteriaceae**	
*Klebsiella pneumoniae*	10 (14.5)
*Enterobacter spp.*	9 (13.0)
*Klebsiella oxytoca*	4 (5.8)
*Klebsiella ozaenae*	3 (4.3)
*Escherichia coli*	3 (4.3)
*Escherichia vulneris*	2 (2.9)
*Citrobacter braakii*	2 (2.9)
*Citrobacter amalonaticus*	2 (2.9)
*Cronobacter spp.*	1 (1.5)
*Escherichia hermannii*	1 (1.5)
*Proteus mirabilis*	1 (1.5)
*Raoultella ornithinolytica*	1 (1.5)
**Moraxellaceae**	
*Acinetobacter baumannii/calcoaceticus*	1 (1.5)
**Yersiniaceae**	
*Serratia ficaria*	1 (1.5)
*Serratia fonticola*	1 (1.5)
*Serratia marcescens*	1 (1.5)
**Erwiniaceae**	
*Pantoea spp.*	14 (20.3)
**Unidentified**	
No matching identification	24 (34.8)

Despite the high contamination burden, we observed low rates of antimicrobial resistance overall: only 2 of the 69 masks harbored at least 1 isolate resistant to antibiotics commonly used for GNB (Table 2). Indeed, resistance rates were high only for erythromycin (59.4%), an antibiotic that is less commonly used for GNB, and were low (<5.0%) for all other antibiotics tested (Table 2). Contamination rates for MRSA and VRE were also low (0 and 1.5%, respectively).

**Table 2.** Resistance Rate for Each Antibiotic for All Isolated Gram-negative Bacteria on All Face Masks^a^

Antibiotic	% Resistant
Ciprofloxacin	1.4
Ceftazidime	0.0
Ceftazidime + Clavulanic Acid	0.0
Meropenem	1.4
Trimethoprim/Sulfamethoxazole	1.4
**Gentamicin**	**1.4**
**Tetracycline**	**4.4**
**Erythromycin**	**59.4**

a Antibiotics are grouped into commonly used to treat gram-negative infection and not commonly used to treat gram-negative infection are shown in bold.

### Analysis of risk factors for HCW mask contamination

**Fig. 1.**
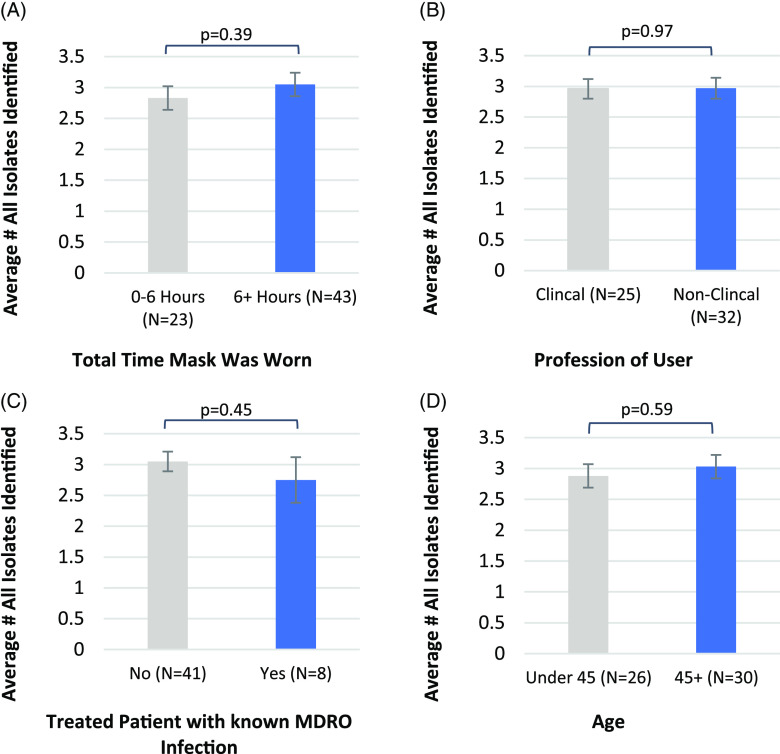
Comparison of face-mask contamination among different HCW donor categories, with standard error bars shown. Average number of all different microorganisms isolated from the mask for (A) masks worn <6 hours, and >6 hours (*P* = .39); (B) clinical versus nonclinical profession of user (*P* = .97); (C) users who did (yes) nor did not (no) treat a patient with an active MDRO infection (*P* = .45); and (D) age of user in years (*P* = .59).

**Fig. 2.**
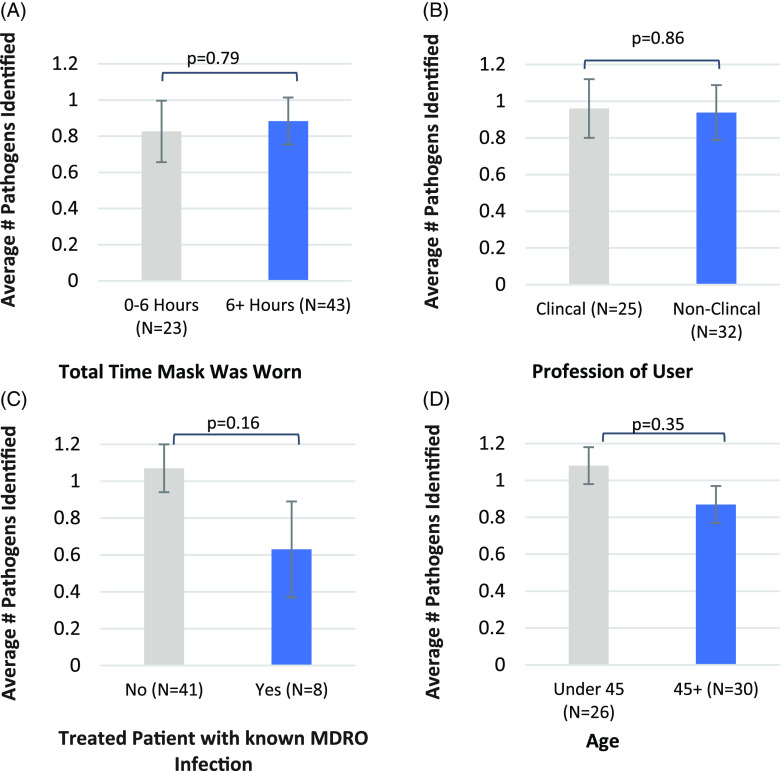
Comparison of face-mask contamination among different HCW donor categories, with standard error bars shown. Pathogens of potential clinical significance include *S. aureus* (*MSSA* and *MRSA*), VRE, *E. coli*, *Enterococcus*, and *K. pneumoniae*. Average number of pathogens isolated from the mask for (A) masks worn <6 hours, and >6 hours (*P* = .79); (B) clinical versus nonclinical profession of user (*P* = .86); (C) users who did (yes) nor did not (no) treat a patient with an active MDRO infection (*P* = .16); and (D) age of user in years (*P* = .35).

Figure 1 compares the numbers of isolates identified from face masks worn by HCWs. Importantly, there were no significant differences in the total number of isolates recovered from masks worn >6 hours versus those worn <6 hours (Fig. 1A). Users with different job functions or different age groups showed no significant differences (Fig. 1B and 1D). Also, whether the user provided care or not to a patient with a known MDRO infection did not result in a significant difference in the number of isolates recovered (Fig. 1C). We analyzed contamination burden specifically with clinically important pathogens. Even in this case, there were no significant differences in the total number of pathogenic isolates for all categories (Fig. 2).

## Discussion

Face masks are a critical tool in the prevention of transmission of respiratory pathogens, and proper use and disposal are paramount to ensuring that they retain maximum effectiveness, especially when assisting the frail, at-risk population in nursing homes. We observed that face masks worn by nursing home personnel were often contaminated with multiple organisms, including potentially pathogenic and antibiotic-resistant bacteria. Prolonged duration of face mask wearing, however, was not associated with increased contamination rates, and neither with profession (clinical vs nonclinical), whether the user provided care to a patient with a known MDRO infection, or age (<45 vs >45 years). Due to relatively limited sample size, it should be noted that while very large differences in contamination burden can be excluded in the target population represented by our sample, smaller differences may still be present that fall beyond the statistical power of our data.

Few studies have addressed bacterial contamination of masks worn for prolonged periods in the nursing home setting. In a study focused on common, clinically important respiratory viruses only, rates did not exceed 10%.^9^ Interestingly, that study also reported increased viral contamination on masks worn >6 hours. In the case of bacteria, our data showed consistent bacterial contamination on HCW masks. When focusing only on clinically important bacteria, rates were lower although not negligible (15.9% for *S. aureus* and 31.9% for gram-negative organisms). In a study on a limited number of healthcare personnel, bacterial contamination was the norm and probably originating mainly from the wearer rather than from the environment.^10^ However, that study was carried out on surgeons in operating rooms, a very different setting from ours. Because prolonged mask wearing in general patient care is a relatively new habit spurred by the necessity of containing severe acute respiratory coronavirus virus 2 (SARS-CoV-2) transmission, there is not enough information available on its impact on mask colonization.

Our data suggest that, at least in our facility, the organisms contaminating HCW masks may be different from those colonizing patients. Recent studies conducted in the same facility have shown endemic high (VRE) or moderate (MRSA) rates of gram-positive pathogens, as well as GNBs resistant to ceftazidime, carbapenem, and especially ciprofloxacin.^11,12^ Conversely, we found no MRSA, only 1 VRE, and very low resistance rates to the aforementioned antibiotics in GNBs among strains isolated from HCW masks.

Our study had strengths and limitations. Our sampling size was dictated more by available resources and by the overall number of HCWs present in the facility and available for mask donation than by statistical power considerations. Indeed, we did not perform sample power calculations because limited data were available for estimating the expected burden of mask contamination in this specific setting and these conditions. We were unable to deternime if and how much certain categories of HCW might be expected to carry a higher bacterial burden on their masks, which is one reason we were interested in investigating this topic. Therefore, establishing reference scenario upon which to base power calculations was challenging. Additionally, we performed screening in a single facility, which may not be representative of the majority of other facilities, and we we were unable to determine whether the number of participants in each category was proportionate to the actual percentage of all facility personnel in that category. Thus, enrollment bias may have been present. Performing larger studies involving multiple facilities and a large sample size would facilitate the discovery of small differences in mask contamination among different categories of HCWs as well as an understanding of whether the observed trends are generalizable.

Among other limitations of our study is the combination of universal masking adopted at the facility and collection at the end of a work shift. These 2 factors led us to obtain a majority of masks worn for a relatively long period of time (between 4 and 8 hours) and few masks worn for a shorter period. Thus, we could not investigate whether bacterial colonization burden gradually increases during the initial few hours a mask is worn. In the aforementioned study on surgeons,^10^ steady increases in bacterial contamination were observed over a set of shorter, 2-hour intervals, suggesting that the maximum contamination rate may have been achieved in <6 hours. Another study reported that masks may progressively lose effectiveness in preventing dispersion of aerosolized bacteria from the wearer’s mouth until becoming ineffective after ∼2 hours.^13^

We were unable to separately assess the inner and outer surfaces of masks due to possible cross contamination at the time the mask was provided to us by the HCWs. The potential clinical significance of testing inner and outer layers is uncertain but should be explored in the future. In some cases, higher bacterial and fungal counts of the same common organisms have been observed on the outer layer in hospital settings.^14^ However, it should be noted that penetration of microorganisms between layers is possible to varying degrees depending on humidity level and specific organisms among other factors.^15^

This study also has a series of strengths, including collecting masks from a representative sample of all the professions typically associated with nursing home care, including HCWs with and without direct contact with patients. Indeed, it is interesting that potential pathogens were found on masks worn by the latter population at a similar rate. This observation, if confirmed in other studies, should inform mask hygiene policy in healthcare environments. Another strength of our study lies in the consistency and appropriateness for the setting of the type of mask worn by all personnel (surgical mask rather than cloth mask, which may differ in the burden and species of bacteria they harbor after prolonged use).^16^ Finally, the identification of microorganisms to species level and antimicrobial resistance profiles provides novel and useful information of clinical relevance, including uncovering potential pathogens that are known for high transmissibility in healthcare settings. Further research should be devoted to developing scientific tools to measure and compare the increased likelihood of an HCW transmitting their own bacteria when not wearing a mask. All factors ranging from frequency of mask touching by the HCW, to the antimicrobial properties of the skin itself^17^ should be considered in these studies.

In conclusion, HCW face masks were consistently colonized with bacteria in our nursing home setting, including potentially pathogenic organisms in a significant number of cases. Attention to appropriate placement of safe mask disposal bins and training in correct donning of masks appears to be of high importance, as is more research to establish how contamination accumulates over time and how often masks should be changed. Additionally, our findings suggest that further research to clarify the possibility, magnitude, and facilitating factors of direct or indirect transmission of microorganism from personnel face masks to patients is warranted.
